# Cutaneous Pseudoepitheliomatous Hyperplasia Secondary to Embedded Canine Pet Hair Mimicking Squamous Cell Carcinoma

**DOI:** 10.1155/crdm/4229969

**Published:** 2026-09-23

**Authors:** Savannah G. Moore, Maxmillian J. Field, Logan S. D’Souza

**Affiliations:** ^1^ Quillen College of Medicine, East Tennessee State University, Johnson City, Tennessee, USA, etsu.edu; ^2^ Brody School of Medicine, East Carolina University, Greenville, North Carolina, USA, unc.edu; ^3^ Forest Dermatology, Mohs Micrographic Surgeon & Dermatologic Oncologist, University of North Carolina School of Medicine-Asheville, Asheville, North Carolina, USA, unc.edu

## Abstract

Although benign, cutaneous pseudoepitheliomatous hyperplasia (PEH) is oftentimes difficult to distinguish from squamous cell carcinoma (SCC). PEH arises from underlying dermal neoplasia, infection, or inflammation. This case report presents the case of a patient with PEH that occurred as a reactive inflammatory response to a foreign body canine hair, which was originally misdiagnosed as SCC. This case highlights a unique etiology of PEH and the importance of using the patient’s social history, physical examination, and appropriate biopsy considerations in complex dermatologic cases where pathology alone is not definitive.

## 1. Introduction

Pseudoepitheliomatous hyperplasia (PEH) is a benign squamous epithelial proliferation that is strongly associated with neoplasia, infection, or inflammation of the underlying dermis [1]. Although benign, PEH is particularly difficult to distinguish from squamous cell carcinoma (SCC) when tissue sampling is limited or when clinicopathological correlation is incomplete, as both diagnoses may demonstrate irregular squamous proliferation and hyperkeratosis on biopsy specimens [1–3]. Cutaneous PEH has been linked to foreign body reactive inflammation, including tattoo ink and orthopedic implant etiologies [4–6]. Foreign body hair can lead to several dermatologic conditions, including hair splinters, cutaneous pili migrans, pilonidal sinuses, and foreign‐body granulomas [7–10]. These conditions are oftentimes more prevalent in cosmetologists and pet groomers due to occupational exposure. Despite the commonality of PEH and its diverse range of foreign body sources, canine hair has not been previously reported as a cause of cutaneous PEH. This case expands the spectrum of known etiologies of PEH and highlights the importance of comprehensive clinical examination, social history assessment, and clinicopathological correlation when evaluating lesions suspicious for malignancy. Furthermore, it illustrates that embedded canine hair may represent a rare but significant mimic of SCC, which can lead to diagnostic uncertainty and potentially unnecessary interventions. This report presents the case of a foreign body canine hair causing PEH in a household pet owner that was originally misdiagnosed as SCC.

## 2. Case Presentation

A 70‐year‐old male presented to dermatology for a chief complaint of a nonhealing lesion on the right foot for six months. Upon initial examination, a 0.7‐cm hypertrophic erythematous papule with hyperkeratotic scale on the right lateral dorsal foot was identified. Shave biopsy showed atypical squamous proliferation worrisome for SCC. The patient returned to clinic 4 weeks later for excision with pathology showing residual SCC with clear margins. The patient returned to clinic 7 months later due to lesion recurrence. Repeat shave biopsy showed transected atypical squamous proliferation with the base unavailable for review; therefore, SCC could not be excluded. Further comments noted associated suppurative inflammation, but the lesional epithelium exhibited greater complexity than typically seen in a reactive process. The patient was referred for Mohs surgery (Figure 1(a)). Microscopic evaluation of the first Mohs stage showed a foreign body suspicious for nonhuman hair. Repeat intraoperative examination revealed several short, coarse dark hairs in the plantar web space of the patient’s feet, and further history revealed that the patient owned a Rottweiler dog (Figure 1(b)). These findings resulted in a new differential diagnosis of PEH due to cutaneous penetration of canine hair rather than SCC. Residual hairs were extracted with a hemostat. Pathology consult confirmed foreign body with reactive inflammatory changes, negative for malignancy, confirming the incidental pet hair as the cause of the cutaneous proliferation (Figure 2(a–c)). Eight weeks later, the patient returned for routine follow‐up; the site was healing appropriately and asymptomatic (Figure 3).

**FIGURE 1:**
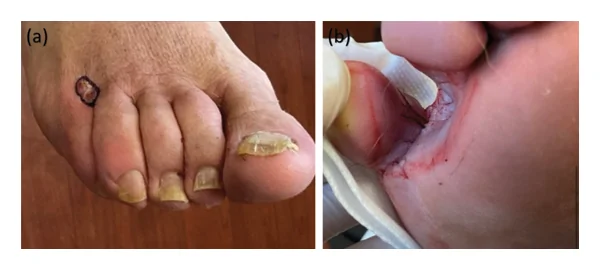
Clinical photographs from Mohs surgical visit. Preoperative: hypertrophic, erythematous papule with hyperkeratotic scale on the right lateral dorsal foot (a). Intraoperative: short, coarse dark hairs in the plantar web space of the patient’s feet (b).

**FIGURE 2:**
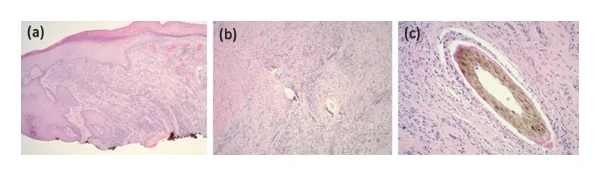
Mohs debulk: Biopsy site changes and continues to show irregular epidermal hyperplasia; no malignant cells (H&E stain; 4× magnification) (a). Mohs en face section: Cross sections of multiple naked hair shafts of varying size and pigment in the dermis with surrounding fibrosis and inflammation; no malignant cells (H&E stain; 4× magnification) (b). Mohs en face section: Longitudinal section of naked pigmented hair shaft with surrounding fibrosis and inflammation; no malignant cells (H&E stain; 10× magnification) (c).

**FIGURE 3:**
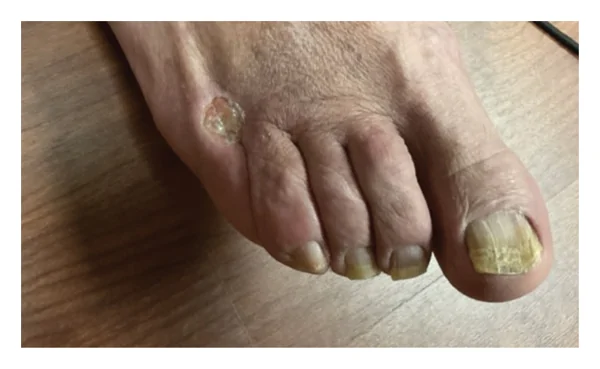
Clinical photograph from 8‐week Mohs postoperative follow‐up visit. Postoperative wound with granulation tissue on the right lateral dorsal foot healing appropriately by secondary intention without complication or sign of lesion recurrence.

## 3. Discussion

This case demonstrates the challenge of distinguishing a hypertrophic, hyperkeratotic papule as either malignant (SCC) or benign (PEH). Several dermatologic processes present with clinical characteristics similar to PEH, adding to the complexity of its clinical diagnosis. Additional considerations for the differential diagnosis of a hypertrophic, hyperkeratotic papule include hypertrophic actinic keratosis, hypertrophic lichen planus, verruca vulgaris, and keratoacanthoma. Histologically, PEH is characterized by thick, elongated, downward epidermal projections that commonly represent expansion of the follicular infundibulum. Projections have variable depth and often extend to the upper to mid reticular dermis. Hypergranulosis and ortho‐ or parakeratosis are oftentimes present. Keratinocytes may show minimal cytologic atypia, mostly within the stratum basale [1].

PEH is commonly a secondary complication to an extensive range of primary conditions, including infections, inflammation, neoplasia, and trauma. Parasitic infections such as leishmaniasis, amebiasis, and enterobiasis can display PEH patterns on areas of the wrists, gastrointestinal tract, and vulva, respectively. PEH can also arise secondary to viral etiologies, such as human papilloma virus and chronic herpes simplex virus [11]. Several cases of PEH induced by foreign body material have been reported, including tattoo ink, permanent makeup, orthopedic implants, and retained bee stinger (Table 1). There are several physical mechanisms that make short, course hairs, such as those from a Rottweiler dog, particularly prone to penetration through the stratum corneum. Due to the concentration of force over a small cross‐section, short hair has rigidity, columnar strength, and resistance to bending under an axial load [12]. Additionally, hairs shed from dog breeds like Rottweilers have tapered or fractured ends, which can act as a needle, reducing the contact area while increasing pressure on the localized region. Lastly, although large amounts of hair are shed, increasing the opportunity for frictional contact, the hairs are very small and easily overlooked. In this case, pet hair can be applied to the Karydakis triad of pilonidal sinus disease [13]. This triad consists of three factors that increase the risk of cutaneous hair penetration: hair properties, force, and skin vulnerability. As seen in this case, the interdigital web space is considered a vulnerable area due to moisture. Although there is a current lack of literature connecting foreign body pet hairs to PEH, this phenomenon provides support for pet hair penetration as a unique etiology of PEH.

**TABLE 1:** Reported cases of PEH secondary to foreign body material within the current literature.

Author (Year)	Age/sex	Foreign body material	Anatomical location	Clinical presentation	Histopathological findings
Hur et al. (1991) [16]	54/M	Retained bee stinger	Location unknown	Ulcerative, erythematous plaques with irregular borders	Epidermal necrosis and marked pseudoepitheliomatous hyperplasia
Balfour et al. (2003) [5]	27/F	Purple tattoo pigment	Location unknown	Verrucous plaques	Marked epidermal hyperplasia with focal keratin‐filled cystic dilations, local mild reactive keratinocytic atypia
Coors et al. (2004) [17]	55/F	Permanent makeup	Upper and lower lip	Hard erythematous nodules	Irregular‐shaped rete ridges extending deep into dermis
Cui et al. (2007) [18]	59/F	Tattoo pigment (red, green, yellow)	Lateral left leg	Granular and pitted pink lesion with well‐demarcated, irregular borders	Pseudoepitheliomatous hyperplasia with irregular acanthosis of epidermis and follicular infundibula, hyperkeratosis, and parakeratosis
Kluger et al. (2008) [19]	Case 1: 30/F Case 2: 32/F Case 3: 54/F	Case 1: red tattoo pigment Case 2: red tattoo pigment Case 3: red tattoo pigment	Case 1: external right heel Case 2: upper back Case 3: heel	Case 1: verrucous lesion Case 2: keratotic verrucous nodule Case 3: firm ulcerated nodule	Case 1: Marked epidermal hyperplasia with epidermal ridges extending deep into reticular dermis without any keratinocyte atypia Case 2: Marked irregular epidermal hyperplasia without atypia Case 3: Typical aspect of PEH associated with infiltration of dermis by lymphocytes and histiocytes without granuloma
de Roeck et al. (2012) [20]	50/F	Red tattoo pigment	Lateral left leg	30‐mm large well‐demarcated tumor and three satellite 10‐mm papules	Irregular marked epidermal hyperplasia with hyperkeratosis and dermal infiltrate of chronic inflammatory cells
Breza et al. (2013) [21]	31/M	Purple tattoo pigment	Right leg	Tender and pruritic plaques	Pseudoepitheliomatous reaction
Tammaro et al. (2014) [22]	26/F	Tattoo pigment (purple, green, blue‐black pigment)	Posterior right leg	Verrucous plaque	Hyperkeratosis, parakeratosis, pseudoepitheliomatous hyperplasia with marked perivascular and periadnexal mononuclear inflammatory infiltrate in dermis and superficial epidermis
Kazlouskaya and Junkins‐Hopkins (2015) [23]	Case 1: 47/M Case 2: 44/F	Case 1: red tattoo pigment Case 2: red tattoo pigment	Case 1: lateral right leg Case 2: location unknown	Case 1: exophytic verrucous plaque Case 2: crusted nodules with excoriations and postinflammatory pigment alteration	Both cases: marked epidermal and infundibular hyperplasia. Biopsy from Case 2 also showed focal lichenoid changes and only rare red pigment granules
Broussard‐Steinberg et al. (2018) [24]	52/F	Red tattoo pigment	Left dorsal hand and left anterior wrist	Firm, verrucous plaques	PEH admixed with dense lichenoid infiltrate with numerous necrotic keratinocytes
Badavanis et al. (2019) [4]	30/F	Red tattoo pigment	Medial aspect of left ankle	Nodule with verrucous and partly erosive surface	Epidermal pseudoepitheliomatous hyperplasia, characterized by irregular acanthosis and parakeratosis
Sauvageau et al. (2019) [25]	73/M	Red tattoo pigment	Inferior, left lateral knee	Verrucous plaque with surrounding inflammation	Marked epidermal hyperplasia and hyperkeratosis with mixed papillomatous/verrucous and endophytic growth patterns.
Kheradmand et al. (2020) [26]	48/F	Purple tattoo pigment	Right lower leg	Scaly and crusted verrucous plaques	Dermal tattoo pigment with overlying PEH
Behera et al. (2022) [27]	21/M	Blue tattoo pigment	Left hand	Erythematous to bluish verrucous plaque	Hyperkeratosis, parakeratosis, pseudoepitheliomatous hyperplasia, spongiosis, and lymphocytic exocytosis
Blum and D’Souza (2022) [6]	22/M	Displaced metallic orthopedic implant	Left lateral malleolus	Hyperkeratotic, verrucous‐appearing plaque	Papillomatous and endophytic squamous proliferation with keratin plug, parakeratin, and mild pleomorphism of keratinocytes extending to the base of the biopsy
Rahbarinejad et al. (2023) [28]	Case 1: 30/M Case 2: 53/F	Case 1: red tattoo pigment Case 2: red tattoo pigment	Case 1: lower leg Case 2: left leg	Case 1: scaly, raised exophytic lesion Case 2: raised keratotic lesions with surrounding erythema	Case 1: orthokeratosis, hyperplastic squamous epithelium, papillomatous change, and lichenoid inflammation at superficial dermis Case 2: Lichenoid‐type inflammatory reaction with pseudoepitheliomatous hyperplasia

The key contributors of this case that led to the definitive diagnosis of PEH were the social history and physical examination findings gathered during the Mohs surgical visit in addition to Mohs histology concerning for foreign body. Several immunohistochemical markers have been studied in differentiating PEH from SCC, including E‐cadherin, matrix metalloproteinases‐1, and p53 [14]. However, despite the advancement of technology in histopathology, the foundational components of the patient history and physical examination still hold diagnostic value, especially in dermatology. As per Zaman, many dermatologic diagnoses are predominantly based on clinical examination features [15]. While pathology plays a significant role in dermatology, it alone was unable to give a definitive diagnosis in this case. The integrative combination of foundational clinical skills and current pathology resulted in the proper diagnosis and treatment plan for this patient. Although oftentimes overlooked, this case also emphasizes the significant role a patient’s social history can hold, particularly in complex cases.

A limitation to the clinical process that potentially resulted in delayed diagnosis, and therefore unnecessary visits and treatment interventions, was a potentially nonideal biopsy method. PEH may be misinterpreted as SCC, especially if a biopsy specimen is superficial and does not contain a sufficient portion of the dermis [1]. When the patient returned to clinic with concern for recurrence after the initial excision, another shave biopsy was performed, which may not have had sufficient depth into the dermis. It was noted from the pathologist that suppurative inflammation was present, which could have led to a differential diagnosis including PEH, but with the history of the lesion previously diagnosed as a malignancy, the lesion was treated as a recurrent SCC. Potentially, a punch biopsy or surgical biopsy would have provided a more comprehensive view of the lesion. However, it is important to acknowledge that each biopsy method is associated with its own limitations. Although a punch biopsy can provide a longitudinally full‐thickness specimen, the small diameter of the punch biopsy tool may inhibit cross‐sectional assessment, possibly requiring multiple sections to view the entire lesion. Regarding excisional biopsy, this method is more laborious, requiring additional suture closure and removal. Complications rates are higher with excisional biopsies, including increased risk of infection, wound dehiscence, and scarring. Considering each biopsy method, punch biopsies appear to be the most appropriate method when diagnosing PEH. Choosing a biopsy tool with a larger diameter may improve cross‐sectional sampling.

As seen in this case, when a lesion diagnosed as recurrent SCC demonstrates unexpected suppurative inflammation on histopathology, these findings should generate a new differential diagnosis. A methodical clinical protocol should be considered when reevaluating the diagnosis. A suggested protocol could include the following: (1) Obtain more social history from the patient, including information about immunosuppression, trauma, animal or environmental exposures, or any other foreign body sources. Regarding lesions on the lower extremities or feet, comorbidities such as diabetes or neuropathy are also pertinent knowledge. (2) Perform a thorough physical examination around the lesion, inspecting for embedded material or other external findings. (3) Rebiopsy the lesion using a large‐diameter punch biopsy method. It may also be helpful to provide the pathologist with a full clinical context. (4) Correlate the clinical data with histopathologic findings to determine a definitive diagnosis. Once a source of inflammation has been identified, treat the underlying cause and follow‐up to confirm resolution.

This is likely the first report of PEH caused by a foreign body pet hair, which occurred in a patient owning a Rottweiler dog. Differentiating between PEH and SCC can be difficult clinically and histopathologically. However, definitive diagnosis is crucial for appropriate intervention. This case highlights the significance of obtaining a comprehensive social history and physical examination in collaboration with dermatopathology, as well as considerations of the biopsy method.

**Nomenclature**
PEH: Pseudoepitheliomatous hyperplasia
SCC: Squamous cell carcinoma

## Funding

No funding was received for this report.

## Disclosure

This work was performed as part of employment of authors at Forest Dermatology, Asheville, NC.

## Consent

Informed consent was obtained from the patient for publication of this report.

## Conflicts of Interest

The authors declare no conflicts of interest.

## Data Availability

The data that support the findings of this study are available from the corresponding author upon reasonable request.

## References

[1] Zayour M. and Lazova R. , Pseudoepitheliomatous Hyperplasia: A Review, The American Journal of Dermatopathology. (2011) 33, no. 2, 112–126, 10.1097/DAD.0b013e3181fcfb47. 21399447

[2] Tan K. B. , Tan S. H. , Aw D. C. et al., Simulators of Squamous Cell Carcinoma of the Skin: Diagnostic Challenges on Small Biopsies and Clinicopathological Correlation, Journal of Skin Cancer. (2013) 2013, 752864–10, 10.1155/2013/752864. PMC3708441 23878739

[3] Chakrabarti S. , Chakrabarti P. R. , Agrawal D. , and Somanath S. , Pseudoepitheliomatous Hyperplasia: A Clinical Entity Mistaken for Squamous Cell Carcinoma, Journal of Cutaneous and Aesthetic Surgery. (2014) 7, no. 4, 232–234, 10.4103/0974-2077.150787. PMC4338470 25722605

[4] Badavanis G. , Constantinou P. , Pasmatzi E. , Monastirli A. , and Tsambaos D. , Late-Onset Pseudoepitheliomatous Hyperplasia Developing Within a Red Ink Tattoo, Dermatology Online Journal. (2019) 25, no. 5, 13030–qt7rk415vm, 10.5070/d3255044072. 31220900

[5] Balfour E. , Olhoffer I. , Leffell D. , and Handerson T. , Massive Pseudoepitheliomatous Hyperplasia: An Unusual Reaction to a Tattoo, The American Journal of Dermatopathology. (2003) 25, no. 4, 338–340, 10.1097/00000372-200308000-00010. 12876493

[6] Blum F. R. and D′Souza L. S. , Cutaneous Pseudoepitheliomatous Hyperplasia from a Displaced Metallic Orthopedic Implant, Case Rep Dermatol Med. (2022) 2022, 10.1155/2022/9139213. PMC9010205 35433057

[7] Salazar C. A. and Gonzalez J. M. , Hidden Agony: Foot Pain Linked to Pet Hair Splinter, Cureus. (2024) 16, no. 6, 10.7759/cureus.63530. PMC11288765 39086785

[8] Brutus J. P. and Chang M. C. , Multiple Pilonidal Sinuses of Both Hands in a Dog Groomer: A Case Report, Hand Surg Rehabil. (2024) 43, no. 1, 10.1016/j.hansur.2023.11.010. 38072305

[9] Räisänen M. P. , Furu H. , Reito A. , and Bindra R. R. , Chronic Occupational Paronychia Secondary to Digital Pilonidal Sinus from Dog Grooming, Journal of Occupational Health. (2025) 67, no. 1, 10.1093/joccuh/uiae076. PMC11827112 39676260

[10] Mairhofer D. , Schmidt P. , Kitzwöugerer M. et al., An Uncommon Zoonosis, Journal der Deutschen Dermatologischen Gesellschaft. (2008) 6, no. 10, 861–864, 10.1111/j.1610-0387.2008.06735.x. 18498380

[11] Delahaye T. , Orduz-Robledo M. , Beltran A. M. et al., Pseudo-Epitheliomatous Hyperplasia and Skin Infections, The Open Dermatology Journal. (2024) 18, no. 1, 10.2174/0118743722304513241001060826.

[12] Bornschlögl T. , Bildstein L. , Thibaut S. et al., Keratin Network Modifications Lead to the Mechanical Stiffening of the Hair Follicle Fiber, Proceedings of the National Academy of Sciences of the United States of America. (2016) 113, no. 21, 5940–5945, 10.1073/pnas.1520302113. PMC4889357 27162354

[13] Gil L. A. , Deans K. J. , and Minneci P. C. , Management of Pilonidal Disease: A Review, JAMA Surgery. (2023) 158, no. 8, 875–883, 10.1001/jamasurg.2023.0373. 37256592

[14] Nayak V. N. , Uma K. , Girish H. C. , Murgod S. , Shyamala K. , and Naik R. B. , Pseudoepitheliomatous Hyperplasia in Oral Lesions: A Review, Journal of International Oral Health. (2015) 7, no. 9, 148–152. PMC4589711 26435636

[15] Zaman J. A. B. , The Enduring Value of the Physical Examination, Medical Clinics of North America. (2018) 102, no. 3, 417–423, 10.1016/j.mcna.2017.12.003. 29650063

[16] Hur W. , Ahn S. K. , Lee S. H. , and Kang W. H. , Cutaneous Reaction Induced by Retained Bee Stinger, The Journal of Dermatology. (1991) 18, no. 12, 736–739, 10.1111/j.1346-8138.1991.tb03166.x. 1806605

[17] Coors E. A. , Wessbecher R. , and von den Driesch P. , Beastly Nodules Instead of Beauty: Pseudoepitheliomatous Hyperplasia Developing After Application of Permanent make-up, British Journal of Dermatology. (2004) 150, no. 5, 10.1111/j.1365-2133.2004.05962.x. 15149522

[18] Cui W. , McGregor D. H. , Stark S. P. , Ulusarac O. , and Mathur S. C. , Pseudoepitheliomatous Hyperplasia- an Unusual Reaction Following Tattoo: Report of a Case and Review of the Literature, International Journal of Dermatology. (2007) 46, no. 7, 743–745, 10.1111/j.1365-4632.2007.03150.x. 17614808

[19] Kluger N. , Durand L. , Minier-Thoumin C. et al., Pseudoepitheliomatous Epidermal Hyperplasia in Tattoos: Report of Three Cases, American Journal of Clinical Dermatology. (2008) 9, no. 5, 337–340, 10.2165/00128071-200809050-00009. 18717610

[20] de Roeck A. , Joujoux J. M. , Fournier F. , Dandurand M. , Meunier L. , and Stoebner P. E. , Florid Pseudoepitheliomatous Hyperplasia Related to Tattoo: A Case Report, International Wound Journal. (2013) 10, no. 5, 539–541, 10.1111/j.1742-481X.2012.01013.x. PMC7950694 22712583

[21] Breza T. SJr, O′Brien A. K. , and Glavin F. L. , Pseudoepitheliomatous Hyperplasia: An Unusual Tattoo Reaction, JAMA Dermatology. (2013) 149, no. 5, 630–631, 10.1001/jamadermatol.2013.2661. 23677107

[22] Tammaro A. , Abruzzese C. , Narcisi A. et al., Localised Pseudoepitheliomatous Hyperplasia: Unusual Cutaneous Reaction Pattern to Tattoo, International Wound Journal. (2016) 13, no. 2, 294–295, 10.1111/iwj.12256. PMC7949587 24720778

[23] Kazlouskaya V. and Junkins-Hopkins J. M. , Pseudoepitheliomatous Hyperplasia in a Red Pigment Tattoo: A Separate Entity or Hypertrophic Lichen Planus- like Reaction?, J Clin Aesthet Dermatol. (2015) 8, no. 12, 48–52. PMC4689511 26705448

[24] Broussard-Steinberg C. , Zemtsov A. , Strausburg M. , Zemtsov G. , and Warren S. , Lichenoid Reaction Pattern with Pseudoepitheliomatous Hyperplasia- a Rare Tattoo Reaction: A Case Report and Review of the Literature, Case Reports in Dermatology. (2018) 10, no. 3, 268–273, 10.1159/000495026. PMC6323364 30631273

[25] Sauvageau A. P. , Mojeski J. A. , Bax M. J. , and Bogner P. N. , Delayed-Onset Pseudoepitheliomatous Hyperplasia Reaction to Red Tattoo Pigment Resembling Squamous Cell Carcinoma, JAAD Case Reports. (2019) 5, no. 3, 222–224, 10.1016/j.jdcr.2018.12.005. PMC6374962 30809565

[26] Kheradmand S. , Perry B. M. , and Bohlke A. , Pseudoepitheliomatous Hyperplasia Arising from Purple Tattoo Pigment, Cutis. (2020) 105, no. 4, E22–E24. 32463858

[27] Behera B. , Kumari R. , Gochhait D. , and Ayyanar P. , Dermoscopy of Pseudoepitheliomatous Hyperplasia Tattoo Reaction Pattern, Dermatology Practical and Conceptual. (2022) 12, no. 3, 10.5826/dpc.1203a121. PMC9464540 36159143

[28] Rahbarinejad Y. , Guio-Aguilar P. , Vu A. N. et al., Pathogenesis, Diagnosis and Management of Squamous Cell Carcinoma and Pseudoepithelial Hyperplasia Secondary to Red Ink Tattoo: A Case Series and Review, Journal of Clinical Medicine. (2023) 12, no. 6, 10.3390/jcm12062424. PMC10056737 36983424

